# Correction: Amin et al. Hippocampal and Cerebellar Changes in Acute Restraint Stress and the Impact of Pretreatment with Ceftriaxone. *Brain Sci.* 2020, *10*, 193

**DOI:** 10.3390/brainsci14090933

**Published:** 2024-09-18

**Authors:** Shaimaa N. Amin, Sherif S. Hassan, Ahmed S. Khashaba, Magdy F. Youakim, Noha S. Abdel Latif, Laila A. Rashed, Hanan D. Yassa

**Affiliations:** 1 Department of Anatomy, Physiology and Biochemistry, Faculty of Medicine, Hashemite University, Zarqa 13133, Jordan; 2 Department of Medical Physiology, Faculty of Medicine, Cairo University, Cairo 11451, Egypt; 3 Department of Medical Education, School of Medicine, California University of Science & Medicine, San Bernardino, CA 82408, USA; 4 Department of Anatomy, Faculty of Medicine, Cairo University, Cairo 11451, Egypt; drmagdy@hotmail.com; 5 Department of Basic Sciences, Riyadh Elm University, Riyadh 12734, Saudi Arabia; ahmedkhashaba@riyadh.edu.sa; 6 Department of Medical Pharmacology, Faculty of Medicine, Cairo University, Cairo 11451, Egypt; noha.gomaa@kasralainy.edu.eg; 7 Department of Biochemistry, Faculty of Medicine, Cairo University, Cairo 11451, Egypt; lailarashed@kasralainy.edu.eg; 8 Department of Anatomy and Embryology, Faculty of Medicine, Beni-Suef University, Beni Suef 62511, Egypt; hanan_yassa2000@yahoo.com

In the original publication [1], the affiliation 1 has been updated. There was also a mistake in Figure 6D, Figure 9D and Figure 10A as published. The errors in Figure 6D, Figure 9D and Figure 10A happened by mistake during the labeling of the photos and plate preparation, processing of the slides, or potentially due to the presence of excess osmic acid stain in the EM photo. Those three photos were replaced and incorporated back into Figure 6, Figure 9 and Figure 10, respectively, under the same labeling. The legends of these figures have not changed. The updated Figure 6, Figure 9 and Figure 10 appear below. The authors state that the scientific conclusions are unaffected. This correction was approved by the Academic Editor. The original publication has also been updated.

## Figures and Tables

**Figure 6:**
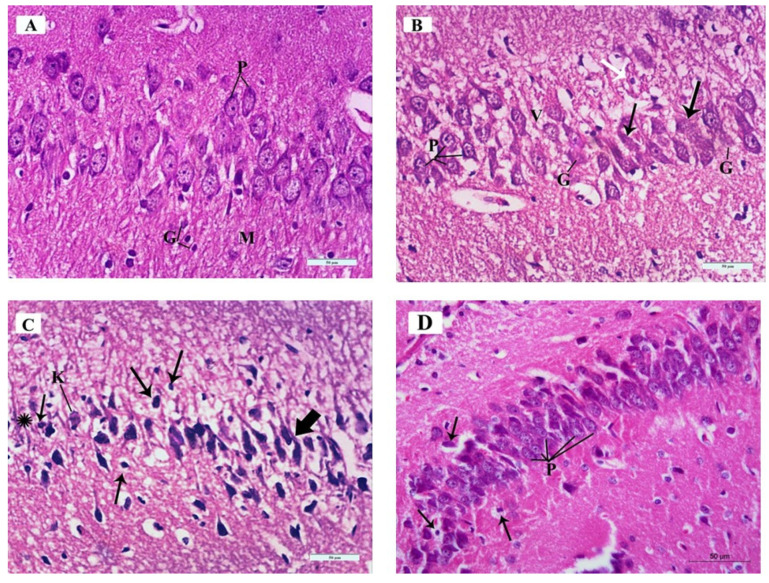
Photomicrographs of Hematoxylin and Eosin (H&E)-stained sections of the mouse hippocampus in the CA3 region of the different study groups: (**A**) Control group and ceftriaxone group; pyramidal cell-layer neurons (P) are uniform in size and evenly arranged. Each neuron has a rounded central vesicular nucleus with prominent nucleolus. The cytoplasm contains prominent basophilic cytoplasmic Nissl’s granules and is surrounded by thin neuropil. The molecular layer (M) contains many glial cells (G) among the neuronal processes. (**B**) ARS group; pleopathologic changes of most of pyramidal neurons’ nuclei as well as vacuolated cytoplasm (V). Some pyramidal cells have vesicular nuclei with clogged marginated chromatin and prominent nucleoli (P); others show pyknosis (white arrow). Few neurons show homogenous nuclei and eosinophilic cytoplasm (arrows) and others have ghost changes (G). (**C**) ARS group; most of the neurons are shrunken, with hyperchromatic nuclei (thick arrows) and vacuolated cytoplasm; others have pyknotic nuclei (thin arrows) or karyolysis (K). Areas devoid of pyramidal neurons (asterisk) are observed. (**D**) ARS + ceftriaxone group; pyramidal neurons are heavily crowded with thin neuropil in between. They have basophilic cytoplasm, well-formed Nissl’s granules, and vesicular nuclei (P). Few cells have pyknotic nuclei with vacuolated cytoplasm (arrows). (Hx. & E. × 400).

**Figure 9:**
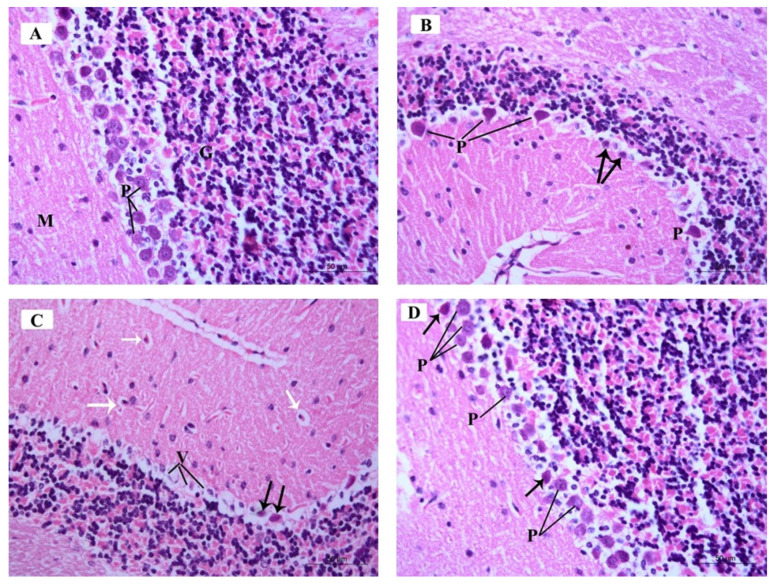
Photomicrographs of H&E-stained sections of the mouse cerebellar cortex of the different study groups: (**A**) Control group and ceftriaxone group display the molecular cell layer (M) containing small scattered basket cells and stellate cells. Purkinje cell layer (P) contains large pyriform-shaped cells having vesicular open-face nuclei and eosinophilic cytoplasm with prominent Nissl’s granules. The granular cell layer (G) contains crowded small deeply stained cells. (**B**) ARS group displays few Purkinje cells (P) with deeply stained nuclei, eosinophilic cytoplasm, and is surrounded by vacuolated neuropil. Other Purkinje cells are shrunken with pyknotic nuclei and vacuolated cytoplasm (arrows). (**C**) ARS group shows Purkinje cells having vacuolated cytoplasm (V) and pyknotic nuclei (black arrows). Basket cells and stellate cells are surrounded with perineuronal spaces (white arrows). (**D**) ARS + ceftriaxone group. Purkinje cells are increased in number; most of them display open-face nuclei (P) while few of them have darkly stained nuclei and eosinophilic cytoplasm (arrow). (Hx. &E. × 400).

**Figure 10:**
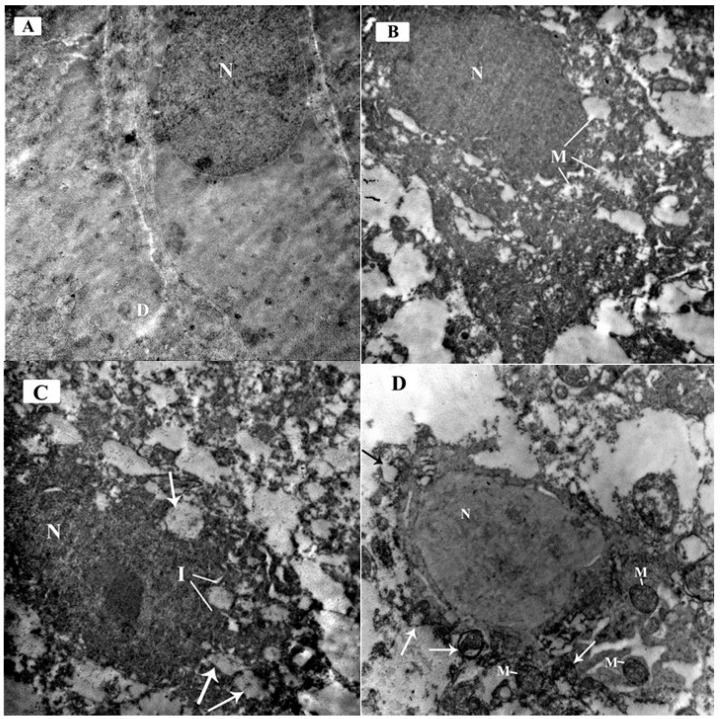
Electron micrographs of the mouse cerebellar cortex in different study groups at the Purkinje cell layer: (**A**) Control group and ceftriaxone group; Purkinje cell has a euochromatic nucleus (N) with fine dispersed chromatin and a well-formed bi-laminar nuclear envelope. The cytoplasm contains scattered cell organelles. A primary dendrite (D) is also seen projecting from the cell membrane. (**B**) ARS group; Purkinje cell have a dark electron-dense nucleus (N) with an irregular nuclear membrane. The cytoplasm contains many swollen mitochondria showing rarified matrix with fragmented or lost cristae (M). (**C**) ARS group displays markedly affected Purkinje cells. The nucleus is shrunken, darkly electron-dense, and irregular in shape with indentation of its nuclear membrane (I). Many dispersed irregularly shaped chromatin aggregates are demonstrated throughout the nucleus. The cell has irregular outline with shrunken cytoplasm containing multiple ballooned mitochondria (arrows) with disrupted cristae. (**D**) ARS + ceftriaxone group; Purkinje cell exhibits a euochromatic nucleus (N) and fine dispersed chromatin. The cytoplasm reveals multiple mitochondria comparable to the control (M) while others are disrupted with separated outer and inner mitochondrial membranes (arrows). (× 6000).
